# *Akkermansia muciniphila* in cardiovascular diseases: opportunities and challenges

**DOI:** 10.3389/fmicb.2026.1786914

**Published:** 2026-06-24

**Authors:** Shihao Li, Chuanmeng Zhang, Min Sha, He Zhu, Ming Chu

**Affiliations:** 1 The Affiliated Hospital of Xuzhou Medical University, Xuzhou Medical University, Xuzhou, China; 2 The Affiliated Taizhou People's Hospital of Nanjing Medical University, Taizhou School of Clinical Medicine, Nanjing Medical University, Taizhou, Jiangsu, China

**Keywords:** *Akkermansia muciniphila*, cardiovascular diseases, clinical translation, gut microbiota, gut-heart axis

## Abstract

Cardiovascular disease (CVD) is one of the leading causes of death worldwide and poses a severe threat to human health. Recent years have witnessed a growing interest in how the gut microbiota regulates the cardiovascular system. *Akkermansia muciniphila* (*A. muciniphila*), a key constituent of this community, has become a focus of research on CVD prevention owing to its critical role in maintaining gut homeostasis, modulating metabolism, and regulating immunity. This review details the beneficial effects and mechanisms of action of *A. muciniphila* in CVD. *A. muciniphila* protects against conditions such as hypertension, atherosclerosis, heart failure, and abdominal aortic aneurysm by repairing the gut barrier, balancing glucose and lipid metabolism, regulating immune-inflammatory responses, and producing protective metabolites such as short-chain fatty acids. However, in pathological states, such as a damaged gut barrier or low-fiber diets, *A. muciniphila* can over-proliferate, accelerate mucus breakdown, and exacerbate inflammation and disease progression—revealing a “double-edged sword” character. Furthermore, diet, medications, and an individual’s baseline gut microbiota directly modulate their abundance, underscoring the need for personalized approaches. Future studies should focus on clarifying strain differences, establishing safe dosing, and optimizing delivery systems to advance the clinical application of *A. muciniphila* in CVD therapy.

## 1 Introduction

Cardiovascular diseases (CVDs), a leading cause of death worldwide by triggering multi-organ dysfunction, account for huge healthcare and societal burdens. Estimates show that CVDs will affect 1.14 billion individuals and cause 35.6 million deaths globally in 2050, displaying a 90.0% increase in the prevalence and a 73.4% increase in the mortality compared to the statistics in 2025 (Chong et al., 2024).

Metabolism is intimately implicated in the progression of CVDs. A growing body of evidence validates an interplay between gut microbiota and the cardiovascular system via the gut-heart axis (Dalman et al., 2025; Snelson et al., 2025). *Akkermansia muciniphila* (*A. muciniphila*), recognized as a next-generation probiotic (Cani et al., 2022), has demonstrated considerable therapeutic potential for gut disorders, metabolic diseases, and cancers (Gao et al., 2025; Ioannou et al., 2025). It is also closely associated with CVDs. The HELIUS (HEalthy LIfe in an Urban Setting) study within the multi-ethnic Dutch cohort has confirmed a causal relationship between the abundance of *A. muciniphila* and the reduced risk of ischemic heart disease (IHD) (Warmbrunn et al., 2024).

In this review, we systematically summarize the research progress on *A. muciniphila* involvement in various CVDs, including hypertension (HTN), atherosclerotic cardiovascular disease (ASCVD), heart failure (HF), and abdominal aortic aneurysm (AAA), as well as their underlying mechanisms. This study aims to provide novel insights into targeted therapies for CVDs by acting on *A. muciniphila*.

## 2 *A. muciniphila* is a rising-star probiotic

*Akkermansia muciniphila*, a gram-negative anaerobic bacterium belonging to the phylum *Verrucomicrobiota*, is named after Anton Akkermans, a famous microbial ecologist. Initially isolated from human feces in 2004, *A. muciniphila* serves as a core commensal bacterium in the vertebrate intestinal mucus layer. It possesses a specialized ecological niche by utilizing mucins as its sole carbon and energy source (Derrien et al., 2004; Desai et al., 2016; Liu et al., 2021). As early as the first month after birth, *A. muciniphila* colonizes the infant’s gut, and its abundance increases significantly within the first year. In adults, the abundance of *A. muciniphila* accounts for 3–4% of the overall gut microbiota (Derrien et al., 2008). Currently, *A. muciniphila* MucT (ATCC BAA-835) is the most intensively studied strain of *A. muciniphila*, showing multiple beneficial properties in enhancing gut barrier function, modulating host immunity, and keeping metabolic balance (Derrien et al., 2017). Amuc_1100, one of the most highly expressed outer membrane proteins in *A. muciniphila*, serves as a major contributor to the therapeutic potential of *A. muciniphila* by mediating Toll-like receptors 2 and 4 (TLR2/4) and the Janus kinase/signal transducer and activator of transcription (JAK/STAT) pathways, without elevating the risks associated with live formulations (Ottman et al., 2017b; Plovier et al., 2017). The Amuc_1100 *A. muciniphila* strain has been authorized as a novel food in the European Union (Turck et al., 2021).

## 3 Mechanistic insights into the effect of *A. muciniphila* on cardiovascular health

**Figure 1:**
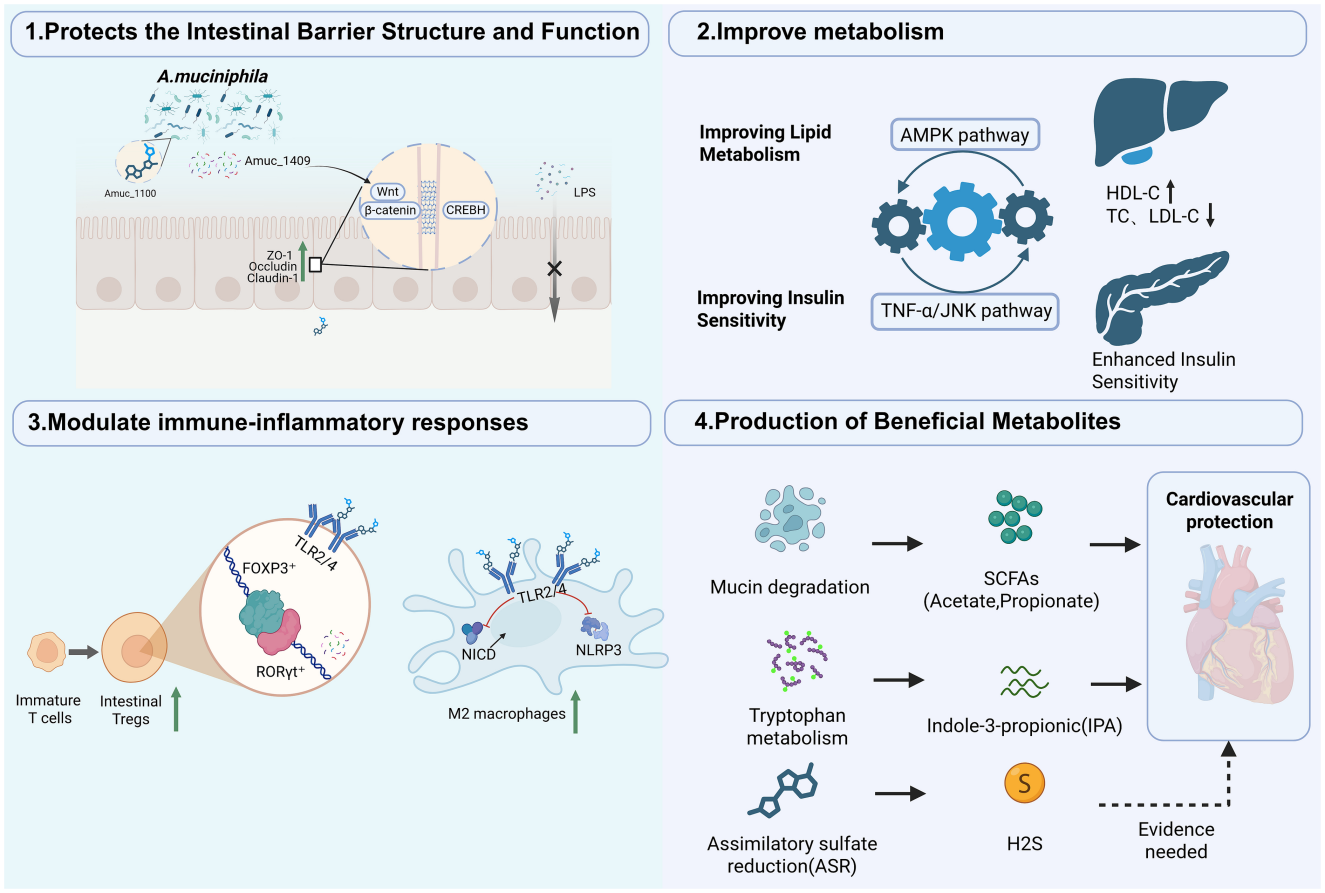
Multifaceted mechanisms by which *A. muciniphila* protects the cardiovascular system. *Akkermansia muciniphila* exerts a protective effect on the cardiovascular system through multiple mechanisms: (1) protects the intestinal barrier structure and function, (2) improves metabolism, (3) modulates immune-inflammatory responses, and (4) produces beneficial metabolites. LPS, Lipopolysaccharide; ZO-1, Zonula occludens-1; CREBH, Cyclic adenosine monophosphate responsive element-binding protein H; Wnt/*β*-catenin, Wingless-related integration site/beta-catenin; FOXP3^+^, Forkhead box P3 positive; MOMA-2^+^ Mφ, Monocyte/macrophage antigen-2-positive macrophages; NICD, Notch intracellular domain; RORγt^+^, Retinoic acid receptor-related orphan receptor gamma t-positive; AMPK, adenosine monophosphate (AMP)-activated protein kinase; TNF-*α*, Tumor necrosis factor-alpha; JNK, c-Jun N-terminal kinase; TLR2/4, Toll-like receptor 2/4; SCFAs, Short-chain fatty acids. HDL-C, High-density lipoprotein cholesterol; LDL-C, Low-density lipoprotein cholesterol; TC, Total cholesterol.

*Akkermansia muciniphila* is beneficial to the cardiovascular system via the gut-heart axis. Specifically, it enhances intestinal barrier function, modulates systemic immunity, offsets inflammatory responses, ameliorates metabolic disorders, and generates beneficial metabolites that directly affect the cardiovascular system. A protective framework involving complicated and intertwined mechanisms allows *A. muciniphila* to prevent and treat CVDs (Figure 1). Figure 1 Multifaceted Mechanisms by Which *A. muciniphila* Protects the Cardiovascular System.

### 3.1 Protects the intestinal barrier structure and function

The intestinal barrier is a critical structure that maintains gut homeostasis, governs selective permeability, and protects against pathogens (Di Tommaso et al., 2021; Turner, 2009). As early as 1997, Anker et al. hypothesized that intestinal congestion and edema impair the function of the intestinal barrier and increase its permeability in patients with chronic heart failure (CHF). This abnormality allows bacterial translocation and endotoxin release into the circulation (e.g., lipopolysaccharide), thereby triggering a systemic inflammatory response via activating the monocyte CD14-TNF-*α* axis and CD14-Toll-like receptor pathways (Anker et al., 1997; Niebauer et al., 1999).

Recent studies have confirmed the multi-dimensional protective effects of *A. muciniphila* in restoring intestinal barrier function. Mechanistically, the transcription factor cyclic adenosine monophosphate responsive element-binding protein H (CREBH) is upregulated by *A. muciniphila*, which further modulates the expression levels of tight junction protein subtypes (e.g., promoting Claudin-5/8 and suppressing Claudin-2). Overexpressed CREBH further facilitates the proliferation and repair of intestinal epithelial cells via the miR-143/145 (microRNA-143/145) signaling axis (Wade et al., 2023). Furthermore, *in vitro* experiments have shown that the culture supernatant of *A. muciniphila* can directly upregulate Occludin and ZO-1, which are known to be capable of sealing gaps in the epithelium, in human intestinal epithelial cells (Li et al., 2016). Beyond these mechanisms, *A. muciniphila* also exerts protective effects through secreting the Amuc_1409 protein, which stimulates intestinal stem cell proliferation and epithelial repair by activating the Wnt/*β*-catenin signaling pathway (Kang et al., 2024; Ma et al., 2025). The protective effects of Amuc_1409 on both the structure and function of the intestinal barrier were consistently robust in various mouse models of radiation injury, chemotherapy (5-FU)-induced enteritis, and natural aging. Importantly, prebiotic interventions (e.g., supplementation of cranberry and platycodon root extract) can enrich *A. muciniphila*, thereby reinforcing the intestinal barrier, mitigating endotoxin translocation, and preventing metabolic abnormalities (Anhe et al., 2017; Luo et al., 2023).

### 3.2 Improve metabolism

Metabolic abnormalities, such as dysglycemia and dyslipidemia, drive the development and progression of CVDs by causing energy supply imbalance, inflammatory responses, oxidative stress, and direct vascular injuries (Jakubiak et al., 2022; Skrha, 2021). Accumulating evidence supports a close association between *A. muciniphila*, improved lipid profiles, and enhanced insulin sensitivity. Lower levels of total cholesterol (TC) and low-density lipoprotein cholesterol (LDL-C) and higher levels of high-density lipoprotein cholesterol (HDL-C) are observed in overweight or obese individuals carrying a higher abundance of *A. muciniphila* (Garcia-Gamboa et al., 2024; Wu et al., 2025). In a dyslipidemia mouse model, *A. muciniphila* significantly ameliorates hypertriglyceridemia by eliminating triglyceride-rich lipoprotein remnants via upregulating hepatic low-density lipoprotein receptor (LDLR) and apolipoprotein E (ApoE) (Shen et al., 2016). *A. muciniphila* protects against insulin resistance by inhibiting tumor necrosis factor-alpha (TNF-*α*)/c-Jun N-terminal kinase (JNK)-mediated serine phosphorylation of insulin receptor substrate-1 (IRS-1) (Luo et al., 2023). The outer membrane protein Amuc_1100, as secreted by *A. muciniphila*, suppresses hepatic lipid oxidation and lipogenesis by activating the adenosine monophosphate (AMP)-activated protein kinase (AMPK) signaling pathway (Zheng et al., 2023). Moreover, *A. muciniphila* effectively reduces hepatic lipid accumulation through upregulating genes involved in fatty acid *β*-oxidation (e.g., carnitine palmitoyl transferase 1A [CPT1A]) and downregulating lipogenic genes (Wang et al., 2021).

### 3.3 Modulate immune-inflammatory responses

Immune cells infiltrate and are activated to trigger and perpetuate a local inflammatory cascade in CVDs. Monocytes/macrophages, by releasing various inflammatory mediators, directly drive disease progression by disrupting tissue homeostasis (Fleetwood et al., 2024; Thackeray et al., 2023). *A. muciniphila* can interact with host TLR2/4 via its outer membrane proteins (e.g., Amuc_1100), waging a defense against inflammatory responses via differentiated RORγt^+^ regulatory T cells (Tregs) (Liu Y. et al., 2022). It also polarizes M2 macrophages toward the anti-inflammatory phenotype, thereby suppressing the pro-inflammatory response mediated through the Notch signaling pathway (Giambra et al., 2025). Additionally, the outer membrane protein Amuc_1100 can inhibit NLR family pyrin domain containing 3 (NLRP3) inflammasome activation (Chen et al., 2022), suppress pro-inflammatory cytokines (e.g., TNF-*α* and IL-6), and activate the anti-inflammatory cytokine IL-10, eventually ameliorating an inflammatory response within the local intestinal microenvironment (Keshavarz et al., 2024). Amuc_1409, secreted from *A. muciniphila*, can be delivered directly into cells, where it maintains a stable expansion and an anti-inflammatory property of Tregs (e.g., release of IL-10) by preventing the degradation of Foxp3 (Xie et al., 2025). Short-chain fatty acids (SCFAs), primarily acetate and propionate, are produced during the metabolism of *A. muciniphila*. They not only exert direct cardiovascular protective effects but also indirectly cooperate with Tregs by creating a favorable condition for their differentiation and functional maintenance (Giambra et al., 2025; Hu et al., 2022; Jena et al., 2025; Schwerdtfeger et al., 2025; Zhang K. et al., 2025).

### 3.4 Production of beneficial metabolites

During the degradation of mucin glycans, *A. muciniphila* produces SCFAs, primarily acetate and propionate(Ioannou et al., 2025a; Jena et al., 2025; Ottman et al., 2017a). A large body of evidence has shown that these SCFAs construct a cardiovascular defense network through multiple pathways—including anti-inflammatory actions, metabolic regulation, vascular protection, and neuromodulation—thereby serving as a critical bridge linking the gut microbiota to heart health (Hu et al., 2022; Mousavi Ghahfarrokhi et al., 2024; Ren et al., 2026). *A. muciniphila* can also metabolize tryptophan to yield indole-3-propionic acid (IPA) (Ao et al., 2026; Lin et al., 2025), which likewise possesses cardiovascular protective properties (Lin et al., 2025; Owumi et al., 2024; Wang Y. C. et al., 2024). Moreover, plasma tryptophan and IPA levels are significantly associated with lower cardiovascular risk and reduced all-cause mortality in patients with coronary artery disease (Li et al., 2022). In addition, members of the *A. muciniphila* phylogroup I (AmI) system group possess a complete assimilatory sulfate reduction (ASR) pathway, enabling the intracellular reduction of sulfate to H₂S and its subsequent incorporation into cysteine synthesis (Becken et al., 2021). H₂S is recognized as a cardioprotective gasotransmitter, and its decreased bioavailability is closely linked to atherosclerosis, myocardial ischemia–reperfusion injury, and heart failure (Hou et al., 2025; Kolluru et al., 2023). However, whether the H₂S generated via this ASR pathway can directly confer such protection remains to be established because direct evidence is lacking.

## 4 Therapeutic roles of *A. muciniphila* in CVDs

**Figure 2:**
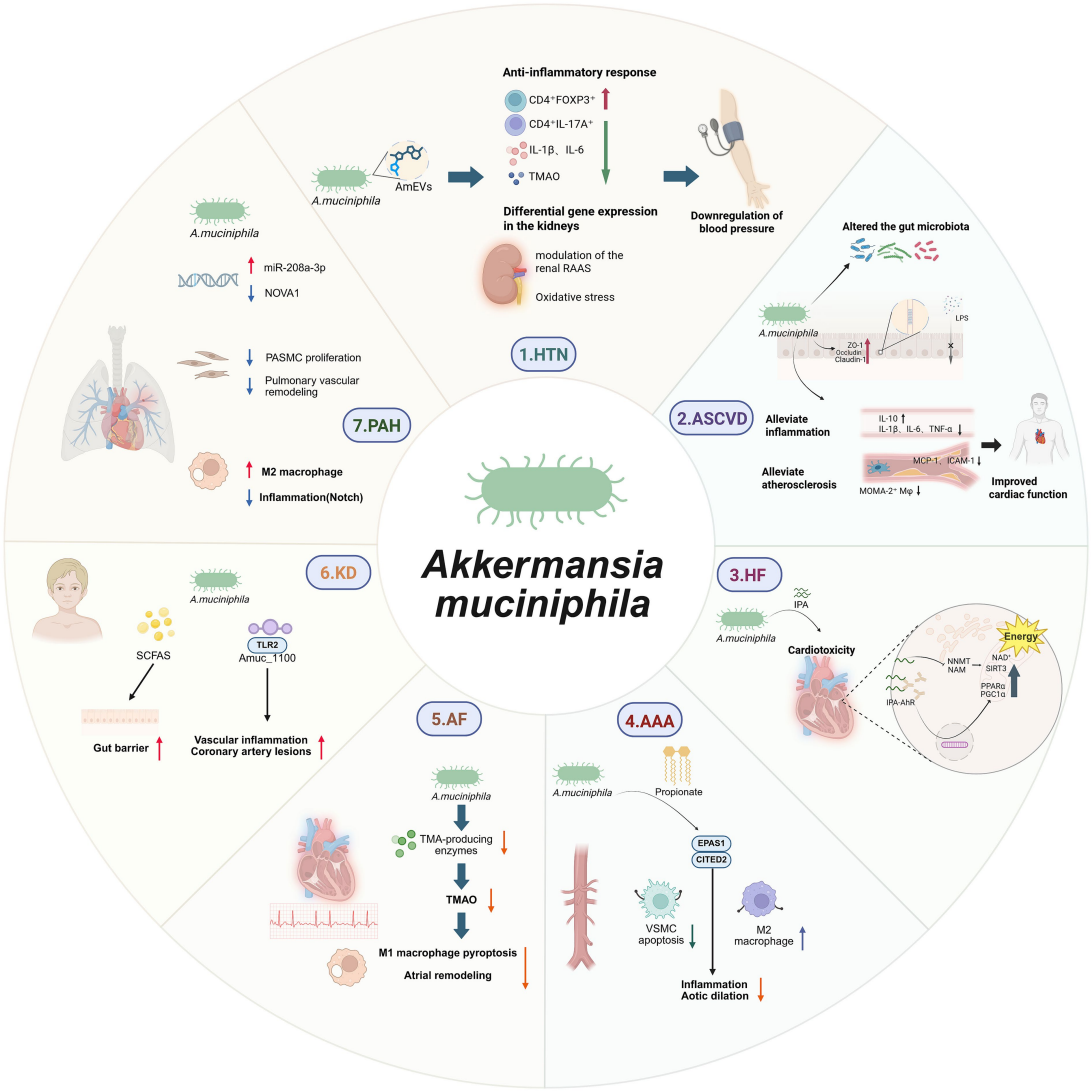
Therapeutic roles of *A. muciniphila* in CVDs. AF, atrial fibrillation; AhR, aryl hydrocarbon receptor; AAA, abdominal aortic aneurysm; AmEVs, *A. muciniphila*-derived extracellular vesicles; ASCVD, atherosclerotic cardiovascular disease; CD4, cluster of differentiation 4; CITED2, CBP/p300-interacting transactivator with ED-rich tail 2; EPAS1, endothelial PAS domain-containing protein 1; FOXP3, forkhead box P3; HF, heart failure; HTN, hypertension; ICAM-1, intercellular adhesion molecule-1; IL-1β, interleukin-1 beta; IL-6, interleukin-6; IL-10, interleukin-10; IL-17A, interleukin-17A; IPA, indole-3-propionic acid; KD, Kawasaki disease; LPS, lipopolysaccharide; MCP-1, monocyte chemoattractant protein-1; miR-208a-3p, microRNA-208a-3p; MOMA-2, monocyte/macrophage antigen-2; NAD+, nicotinamide adenine dinucleotide; NAM, nicotinamide; NOVA1, neuro-oncological ventral antigen 1; PAH, pulmonary arterial hypertension; PASMC, pulmonary artery smooth muscle cell; PGC1*α*, peroxisome proliferator-activated receptor gamma coactivator 1-alpha; PPARα, peroxisome proliferator-activated receptor alpha; RAAS, renin-angiotensin-aldosterone system; SCFAs, short-chain fatty acids; SIRT3, sirtuin 3; TLR2, Toll-like receptor 2; TMA, trimethylamine; TMAO, trimethylamine N-oxide; TNF-α, tumor necrosis factor alpha; VSMC, vascular smooth muscle cell; ZO-1, zonula occludens-1.

To systematically outline the supporting evidence, Table 1 compiles findings from animal models that elucidate potential mechanisms, whereas Table 2 reviews observational and interventional studies in human populations. Figure 2 illustrates the therapeutic mechanisms of *A. muciniphila* in various cardiovascular diseases.

**Table 1:** A summary of studies supporting the therapeutic potential of *A. muciniphila* in animal models.

Disease	Animal model	*A. muciniphila* strain	Formulation	Route of administration	Dosage	Frequency	Duration	Benefits
HTN	SHRs and WKYs (aged 4 weeks)	ATCC BAA-835, DSM 22959, KCTC 15667	AmEVs	Intravenous injection	1.0 × 10^8^ or 1.0 × 10⁹ particles/kg	Once weekly	4 weeks	AmEVs alleviate hypertension in SHRs by reducing IL-1β/IL-6 levels, modulating the Th17/Treg balance, and inhibiting STAT3 phosphorylation. (Kim et al., 2024)
HTN	SHRs and WKYs (aged 4 weeks)	ATCC BAA-835, DSM 22959, KCTC 15667	AmEVs	Intravenous injection	1.0 × 10^8^ or 1.0 × 10⁹ particles/kg	Once weekly	4 weeks	AmEVs protect against hypertension by upregulating renal Agt, At1ar, At2r, Mas1, and Nos2 without increasing the risk of adverse events. (Olarinoye et al., 2024)
HTN	SHRs and WKYs (aged 6–8 weeks)	BNCC 341917	Live *A. muciniphila*	Oral gavage	1.0 × 10⁹ CFU/day	Three times a week	2 weeks	Oral gavage of live *A. muciniphila* reduces plasma levels of renin, aldosterone, ACTH, angiotensin II, cortisol, TMAO, and LPS; increases butyrate levels; and decreases blood pressure. (Yun et al., 2025)
PIH	C57BL/6 J mice (aged 6–8 weeks)	ATCC BAA-835	Pasteurized *A. muciniphila*	Oral gavage	1 × 10^8^ CFU/mL and 5 × 10^8^ CFU/mL	Once daily	2 weeks	*A. muciniphila* prevents mitochondrial dysfunction-mediated apoptosis by activating the PI3K/Akt pathway (Wang H. et al., 2025)
PIH	Adult pregnant Wistar rats (aged 8–10 weeks)	–	*A. muciniphila* suspensions	Oral gavage	2 mL	Once daily	12 days	*A. muciniphila* and propionate/butyrate ameliorate preeclampsia by stimulating spiral artery remodeling and autophagy-driven M2 polarization. (Jin et al., 2022)
ASCVD	ApoE^−/−^ mice on a C57BL/6 J background (aged 8 weeks)	ATCC BAA-835	Live and heat-killed *A. muciniphila*	Oral gavage	5 × 10⁹ CFU/200 μL	Once daily	8 weeks	*A. muciniphila* ameliorates aortic and systemic inflammation in Western diet–fed ApoE-/-mice by reducing intestinal permeability and inhibiting the translocation of gut-derived LPS. (Li et al., 2016)
ASCVD	ApoE^−/−^ mice on a C57BL/6 J background (aged 6 weeks)	ATCC BAA-835	Heat-killed *A. muciniphila*	Oral gavage	1 × 10⁹ CFU/day	Once daily	12 weeks	*A. muciniphila* attenuates atherosclerotic lesion formation via improving dyslipidemia, enhancing cardiac function, rebalancing IL-6/IL-10, and enriching beneficial *Lactobacillaceae* in ApoE^−/−^ mice. (Xiao et al., 2024)
DIC	C57BL/6 J mice (aged 8 weeks, weighed 18–25 g)	*A. muciniphila* (Cat. B336076)	Live and pasteurized *A. muciniphila*	Oral gavage	1 × 10^10^ CFU/100 μL	Twice a week	4 weeks	*A. muciniphila* increases circulating IPA levels to attenuate DOX-induced diastolic dysfunction, cardiac fibrosis, and mitochondrial bioenergetics disorder.(Lin et al., 2025)
AAA	C57BL/6 J mice (aged 5–6 months)	ATCC-BAA-865, DSM 22959	Live and pasteurized *A. muciniphila*	Oral gavage	2 × 10^8^ CFU/180 μL	Once daily	4 weeks	*A. muciniphila* inhibits AAA formation and alleviates tissue injury by restoring gut microbiota diversity, modulating peripheral immunity, and regulating functions of *E. coli*, *Clostridium*, and *Lactobacillus*. (He et al., 2022)
AAA	Male ApoE^−/−^ mice (aged 8 weeks)	ATCC	Live *A. muciniphila*	Oral gavage	2 × 10^8^ CFU/180 μL	Once daily	4 weeks	*A. muciniphila* attenuates AAA development by suppressing macrophage inflammation and VSMC apoptosis via activation of the EPAS1/CITED2 axis (Wang S. et al., 2024)
AF	Male SD rats (200-250 g)	ATCC BAA-835	Live and pasteurized *A. muciniphila*	Oral gavage	1 × 10⁹ CFU/200 μL	Once daily	2 weeks	Oral supplementation with *A. muciniphila* ameliorates cold exposure-induced AF susceptibility by restraining TMA/TMAO production. (Luo et al., 2022b)
KD	SPF WT and Il1r1^−/−^ C57BL/6 J mice (aged 4 weeks)	ATCC BAA-835	Live and pasteurized *A. muciniphila*	Oral gavage	2 × 10^8^ CFU/200 μL	Once daily	2 weeks	*A. muciniphila* attenuates KD-related cardiovascular inflammation by enhancing gut barrier biomarkers, which rely on its component Amuc_1100 through TLR-2 signaling. (Jena et al., 2025)
PAH	Male C57BL/6 mice (aged 6–8 weeks)	ATCC BAA-835	Live *A. muciniphila*	Oral gavage	2 × 10^8^ CFU/200 μL	Once daily	3 weeks	*A. muciniphila* improves hypoxia-induced PIH by regulating the miR-208a-3p/NOVA1 axis. (Bao et al., 2023)

AAA, Abdominal Aortic Aneurysm; ACTH, Adrenocorticotropic Hormone; AF, Atrial Fibrillation; Agt, Angiotensinogen; Akt, Ak strain transforming; AmEVs, *A. muciniphila* Extracellular Vesicles; *A. muciniphila*, *Akkermansia muciniphila*; ApoE^−/−^, Apolipoprotein E-Deficient; ASCVD, Atherosclerotic Cardiovascular Disease; ATCC, American Type Culture Collection; At1ar, Angiotensin II Receptor Type 1a; At2r, Angiotensin II Receptor Type 2; BNCC, BeNa Culture Collection; C57BL/6 J, C57 Black 6; CFU, Colony-Forming Unit; CITED2, Cbp/p300-interacting transactivator with Glu/Asp-rich carboxy-terminal domain 2; DIC, Doxorubicin-induced Cardiotoxicity; DOX, Doxorubicin; DSM, Deutsche Sammlung von Mikroorganismen und Zellkulturen; *E. coli*, *Escherichia coli*; EPAS1, Endothelial PAS domain-containing protein 1; HTN, Hypertension; IL-1β, Interleukin-1 beta; IL-6, Interleukin-6; IL-10, Interleukin-10; Illr1, Immune-related, Lectin-like receptor 1; IPA, Indole-3-propionic acid; KD, Kawasaki Disease; KCTC, Korean Collection for Type Cultures; LPS, Lipopolysaccharide; Mas1, Mas receptor; Nos2, Nitric Oxide Synthase 2; NOVA1, NOVA alternative splicing regulator 1; PAH, Pulmonary Arterial Hypertension; PI3K, Phosphoinositide 3-Kinase; PIH, Pregnancy-Induced Hypertension; RAAS, Renin-Angiotensin-Aldosterone System; SD, Sprague–Dawley; SHRs, Spontaneously Hypertensive Rats; SPF, Specific Pathogen-free; STAT3, Signal Transducer and Activator of Transcription 3; Th17, T helper 17; TMA, Trimethylamine; TMAO, Trimethylamine N-Oxide; TLR-2, Toll-Like Receptor 2; Treg, Regulatory T; VSMC, Vascular Smooth Muscle Cell; WKYs, Wistar-Kyoto rats; WT, Wild-Type.

**Table 2:** Summary of studies supporting the therapeutic potential of *A. muciniphila* in human CVD.

Disease	Population	Sample detection method	Sample size	*A. muciniphila* abundance in diseased populations	References
HTN	Chinese	Metagenomic sequencing	99 HTN patients and 41 healthy controls	Decreased	Li et al. (2017)
HTN	Australian	16S rRNA sequencing	29 HTN patients and 32 healthy controls	No significant change	Nakai et al. (2021)
PIH	Chinese	16S rRNA sequencing	67 PE patients and 85 healthy controls	Decreased	Chen et al. (2020)
PIH	Chinese	Metagenomic sequencing	35 PIH patients (20 with GH and 15 with PE) and 35 healthy controls	Decreased	Lin et al. (2022)
PIH	Chinese	16S rRNA sequencing	92 PE patients and 86 healthy controls	Decreased	Jin et al. (2022)
ASCVD	Chinese	Metagenomic sequencing	188 ASCVD patients and 173 healthy controls	Decreased	Xiao et al. (2024)
ASCVD	Chinese	Metagenomic sequencing	218 ASCVD patients and 187 healthy controls	Decreased	Jie et al. (2017)
HF	Iran	qPCR	20 HFrEF patients and 40 healthy controls	No significant change	Mirhosseini et al. (2025)
HF	Australia	16S rRNA sequencing	26 HFpEF patients and 67 healthy controls	Increased	Beale et al. (2021)
KD	Chinese	Metagenomic sequencing	96 KD patients and 62 healthy controls	Decreased	Han et al. (2024)
AF	Chinese	16S rRNA sequencing	103 winter AF patients and 106 summer AF patients (as controls)	Decreased	Luo et al. (2022b)

AF, Atrial Fibrillation; AmEVs, *A. muciniphila* Extracellular Vesicles; *A. muciniphila*, *Akkermansia muciniphila*; ASCVD, Atherosclerotic Cardiovascular Disease; GH, Gestational Hypertension; HF, Heart Failure; HFpEF, Heart Failure with preserved Ejection Fraction; HFrEF, Heart Failure with reduced Ejection Fraction; HTN, Hypertension; KD, Kawasaki Disease; PE, Preeclampsia; PIH, Pregnancy-Induced Hypertension; qPCR, Quantitative Polymerase Chain Reaction; rRNA, ribosomal RNA.

### 4.1 Hypertension

Primary hypertension is driven by a complicated etiology, including the abnormally activated renin-angiotensin-aldosterone system (RAAS), immune dysregulation, increased excitability of the sympathetic nervous system, and disturbances in water-electrolyte metabolism (DeLalio et al., 2020; Sánchez-Lozada et al., 2023). 16S rRNA sequencing data demonstrate an association between a reduced abundance of *A. muciniphila* and an increased risk of hypertension (Li et al., 2017). Interestingly, the reduction of *A. muciniphila* in spontaneously hypertensive rats (SHRs) is significantly restored by an eight-week exercise intervention to basically the same level as that in normotensive rats. This beneficial effect is further validated via fecal microbiota transplantation (FMT), suggesting that exercise-enriched *A. muciniphila* can directly relieve hypertension (Li et al., 2025). Intravenous administration of *A. muciniphila* extracellular vesicles (AmEVs) significantly lowered systolic blood pressure in SHRs through dual mechanisms. First, AmEVs stimulate the remodeling of immune homeostasis and the suppression of vascular inflammation by interrupting the IL-1β/IL-6-STAT3 inflammatory axis, activating anti-inflammatory Treg cells, and inactivating pro-inflammatory Th17 cells (Kim et al., 2024). Second, they balance the classical and non-classical pathways of RAAS to achieve an antihypertensive effect (Olarinoye et al., 2024). AmEVs also activate pro-oxidant genes *in vivo* without inducing pathological damage or significant adverse effects. Therefore, AmEVs are a potential therapeutic target for hypertension (Kim et al., 2024; Olarinoye et al., 2024), though the safety of chronic administration requires further investigation.

Besides vesicle-based therapies, direct supplementation with live *A. muciniphila* also has a significant antihypertensive effect. Yun et al. (2025) have suggested that a three-week oral *A. muciniphila* supplementation in patients with refractory hypertension remarkably increases its abundance in the gut, accompanying remarkable reductions in systolic and diastolic blood pressure, RAAS activity, and plasma levels of N-oxide (TMAO) and lipopolysaccharide (LPS), as well as elevations in beneficial metabolites (e.g., butyrate). These findings suggest that multiple synergistic mechanisms are involved in the antihypertensive effects of *A. muciniphila*. However, a multicenter study has reported an inconsistent finding that the abundance of *A. muciniphila* is similar between hypertensive patients and healthy controls, although the former exhibit alterations in specific bacterial genera (e.g., increased *Acidaminococcus* and decreased *Ruminococcus*) and microbial metabolic pathways (Nakai et al., 2021). The controversial microbiota-hypertension association can be attributed to variations across populations, disease stages, and methodological approaches.

Pregnancy-induced hypertension (PIH), a condition of elevated blood pressure during pregnancy or in recently new mothers, encompasses a spectrum of disorders such as gestational hypertension and preeclampsia (PE) (Kintiraki et al., 2015). Women with PE usually face a significantly higher risk of developing future cardiovascular diseases (Brown et al., 2013). As a result, regular monitoring of blood pressure and early interventions are critical to improve the long-term outcomes of PIH. Compared to normotensive pregnant women, a significantly lower abundance of *A. muciniphila* is detected in PE patients (Chen et al., 2020; Jin et al., 2022; Lin et al., 2022). Supplementation with short-chain fatty acids (e.g., propionate), known as *A. muciniphila* products, significantly reduces blood pressure and ameliorates clinical symptoms and pathological alterations in rats with PE (Jin et al., 2022). Notably, pasteurized *A. muciniphila* also demonstrated definite therapeutic efficacy against PE, with a high clinical safety profile (Wang H. et al., 2025).

### 4.2 Atherosclerotic cardiovascular disease (ASCVD)

ASCVD, as the leading cause of death among urban and rural residents in China, is closely associated with metabolic risk factors such as obesity, dyslipidemia, and diabetes (Libby et al., 2019). Both clinical and preclinical studies have consistently demonstrated that *A. muciniphila* maintains a very low abundance in individuals with obesity, insulin resistance, and metabolic endotoxemia. Interventions with viable *A. muciniphila* significantly restore normal glucose and lipid metabolism, enhance intestinal barrier function, and attenuate inflammatory responses (Derrien et al., 2017; Liu et al., 2024). A meta-analysis has demonstrated that the anti-atherosclerotic benefit of dietary components rich in polyphenols (e.g., bilberries and blackberries) and alkaloids (e.g., berberine) can be attributed to the elevated abundance of *A. muciniphila*, indicating the protective effect of *A. muciniphila* against ASCVD (Khalili et al., 2023). Metagenomic sequencing data reveal a markedly higher abundance of *A. muciniphila* in the fecal microbiota of healthy controls than ASCVD patients (Jie et al., 2017; Xiao et al., 2024). *In vivo* experiments consistently prove the efficacy of orally viable *A. muciniphila* supplements in treating atherosclerosis in apolipoprotein E-deficient (ApoE^−/−^) mice via relieving aortic plaque burden, dyslipidemia (manifested as a reduced LDL-C/HDL-C ratio), and inflammatory responses (manifested as decreased IL-6 and increased IL-10 levels) (Xiao et al., 2024). Conversely, oral gavage with *A. muciniphila* in ApoE^−/−^ mice fails to mitigate high-fat diet (HFD)-induced hypercholesterolemia and produces no significant alterations in fasting glycemia or glucose tolerance (Li et al., 2016). The precise role of *A. muciniphila* in the regulation of lipid and glucose homeostasis requires further investigation.

Emerging evidence suggests that the therapeutic efficacy of *A. muciniphila* in ASCVD is critically determined by its viability (Li et al., 2016; Xiao et al., 2024), posing potential challenges to clinical safety. As a result, administration strategies for *A. muciniphila* should be optimized to realize effective and safe treatment of ASCVD.

### 4.3 Heart failure (HF)

Heart failure (HF), a clinical syndrome characterized by diminished cardiac output and systemic congestion following structural or functional impairment of the heart, manifests as the terminal phase of diverse cardiac pathologies involving complicated mechanisms. A “gut hypothesis” has been proposed to delineate the mechanistic role of the gut microbiota in HF progression (Nagatomo and Tang, 2015). *A. muciniphila* administration markedly enhances left ventricular ejection fraction (LVEF) and left ventricular fractional shortening (LVFS), reduces serum cardiac injury biomarkers (e.g., lactate dehydrogenase [LDH] and creatine kinase-MB isoenzyme [CK-MB]), and alleviates myocardial injury, atrophy, and fibrosis in a murine model of doxorubicin-induced cardiotoxicity (DIC). This cardioprotective role can be explained by the binding of IPA, as produced by *A. muciniphila*, to the cardiac aryl hydrocarbon receptor (AhR), which further improves mitochondrial bioenergetic metabolism through activating the PPAR*α* signaling pathway (Ghaderi et al., 2022; Lin et al., 2025). Moreover, the interaction of IPA with AhR increases the NAD^+^/NADH ratio and nicotinic acid by upregulating SIRT3 and downregulating nicotinamide N-methyltransferase (NNMT), ultimately ameliorating cardiac injury. Additionally, IPA directly inhibits inflammatory genes and oxidative stress in myocardial tissue, as well as plasma endotoxins (Wang Y. C. et al., 2024). These findings collectively suggest the potential benefits of *A. muciniphila* in treating HF, although its efficacy remains to be explored further.

A markedly increased abundance of *A. muciniphila* in fecal specimens (linear discriminant analysis [LDA] score>3.0) serves as a discriminative microbial feature in patients with preserved ejection fraction (HFpEF), although this population lacks a detailed assessment of intestinal barrier function (e.g., circulating endotoxin concentrations and intestinal permeability) (Beale et al., 2021). Considering the predominant role of *A. muciniphila* in degrading the intestinal mucus layer, an abnormal elevation of its abundance implies a potential disruption of intestinal barrier integrity. However, inconsistent findings show that HFrEF is not associated with the abundance of *A. muciniphila* but is significantly related to vitamin D insufficiency (Mirhosseini et al., 2025). This discrepancy can be attributed to the limited sample size (20 vs. 26), different bacterial quantification methods (qPCR vs. 16S rRNA gene sequencing), and intrinsic pathophysiological heterogeneity across HF subtypes. In the future, multicenter, large-scale cohort studies on FMT and metabolomic profiling are needed to precisely determine the potential causal relationship between *A. muciniphila* and HF.

### 4.4 Abdominal aortic aneurysm (AAA)

The abundance of *A. muciniphila* is reduced in a murine Abdominal Aortic Aneurysm (AAA) model, indicating a potential protective effect against AAA (Xiao et al., 2023). Exogenous administration of *A. muciniphila* alleviates AAA by restoring gut microbial diversity, manifesting as elevated *α*-diversity and abundance of the *Lachnospiraceae* NK4A136 group, enhanced microbiota metabolic capabilities (e.g., the L-rhamnose degradation pathway), and suppressed systemic inflammation (He et al., 2022). Mechanistic investigations further demonstrated that propionate, a metabolite derived from *A. muciniphila*, activates the transcription factor Endothelial PAS domain-containing protein 1 (EPAS1) to bind to the Cbp/p300-interacting transactivator 2 (CITED2) promoter and enhance its transcriptional activity. This cascade at the cellular level eventually inhibits the apoptosis of vascular smooth muscle cells (evidenced by the upregulated SM22α/α-SMA and downregulated cleaved-caspase-3) and promotes M2 macrophage polarization to expand anti-inflammatory properties (evidenced by an increased number of CD206^+^/Arg1^+^ cells). Collectively, these actions synergistically mitigate inflammatory infiltration of the vascular wall, degradation of elastic fibers, and dilation of the aorta (Wang S. et al., 2024). Through the gut microbiota-immune-EPAS1/CITED2 axis, *A. muciniphila* forms a hierarchical regulatory network to act on AAA via probiotic-based interventions. Nevertheless, existing evidence supporting the beneficial role of *A. muciniphila* in AAA is solely sourced from animal experiments, and dynamic alterations in its intestinal abundance and pertinent correlations with AAA remain to be explored in human cohorts.

### 4.5 Atrial fibrillation (AF)

*A. muciniphila* supplementation downregulates key trimethylamine (TMA)-synthesizing enzymes (e.g., CntA/B, CutC/D, YeaW/X) in the gut microbiota of a cold-induced AF model, thereby inhibiting TMA production and the plasma level of its oxidative metabolite, TMAO, consequently suppressing M1 macrophage pyroptosis and atrial structural remodeling (Luo et al., 2022b). However, research on the precise regulation of *A. muciniphila* in AF is lacking, restricting its potential therapeutic application and translational value.

### 4.6 Kawasaki disease (KD)

KD, a rare disease mostly affecting children under 5 years of age, is a condition of acute systemic inflammation and a predominant cause of acquired childhood heart disease in developed countries (Jone et al., 2024). This may cause the development of coronary artery aneurysms. Both genetic predisposition and dysregulated immune activation are involved in the pathogenesis of KD (Beltran et al., 2023). Gut microbiota dysbiosis has also been validated as a pivotal contributing factor to KD. Metagenomic analyses have revealed that the abundance of *A. muciniphila* decreases significantly in the gut of children with KD, which is further associated with resistance to intravenous immunoglobulin (IVIG) therapy and the development of coronary artery lesions (Han et al., 2024). In a mouse model of vasculitis induced by *Lactobacillus casei* cell wall extract (LCWE), gut microbiota dysbiosis, with a typical manifestation of an increased abundance of pro-inflammatory bacteria and a decreased abundance of anti-inflammatory bacteria, drove the progression of vascular inflammation. Oral supplementation with viable or pasteurized *A. muciniphila* and *Faecalibacterium prausnitzii* (*F. prausnitzii*) attenuates cardiovascular pathology via dual mechanisms. From one perspective, SCFAs (e.g., acetate and propionate) synthesized by *A. muciniphila* and *F. prausnitzii* potentiate intestinal barrier function. Simultaneously, the Amuc_1100 protein from *A. muciniphila* directly preserves intestinal epithelial integrity and antagonizes IL-1β-mediated epithelial injuries by activating the Toll-like receptor 2 (TLR-2) signaling pathway (Jena et al., 2025). Collectively, *A. muciniphila* exerts a core protective role in KD-induced vasculitis, providing an avenue for designing adjunctive KD therapeutics.

### 4.7 Pulmonary arterial hypertension (PAH)

PAH is defined by progressively elevated pulmonary vascular resistance and right ventricular dysfunction and can lead to right ventricular dilation, HF, and even death (Bordan et al., 2024). Hypoxia, as a vital etiological factor, induces pathogenic pulmonary vascular remodeling by stimulating aberrant proliferation of pulmonary arterial smooth muscle cells (PASMCs) (Li et al., 2021). By upregulating miR-208a-3p and further targeting the downregulation of NOVA1, *A. muciniphila* displays an inhibitory effect against hypoxia-induced gut microbiota dysbiosis via inhibiting the proliferation of PASMCs and arresting cell cycle progression into the G1/S phase. Consequently, *A. muciniphila* activates the gut-lung axis, reverses pulmonary vascular remodeling, and protects against right ventricular dysfunction, but it does not significantly affect the proliferation of pulmonary arterial fibroblasts (Bao et al., 2023). Notably, *A. muciniphila* triggers M2 macrophage polarization toward the anti-inflammatory phenotype by activating TLR2 and TLR4 signaling pathways, thereafter inhibiting the Notch-mediated pro-inflammatory response (Giambra et al., 2025). The Notch signaling pathway, indispensably involved in the pivotal stage of neointimal formation, shows a potent role in aggravating PAH in murine models (Steffes et al., 2020). This offers indirect evidence that *A. muciniphila* influences the pathogenesis and progression of PAH by inhibiting the Notch signaling pathway. A direct, precise interaction between *A. muciniphila* and Notch signaling in PAH requires further investigation, which may provide novel therapeutic targets for PAH.

## 5 *A. muciniphila* as a double-edged sword

**Figure 3:**
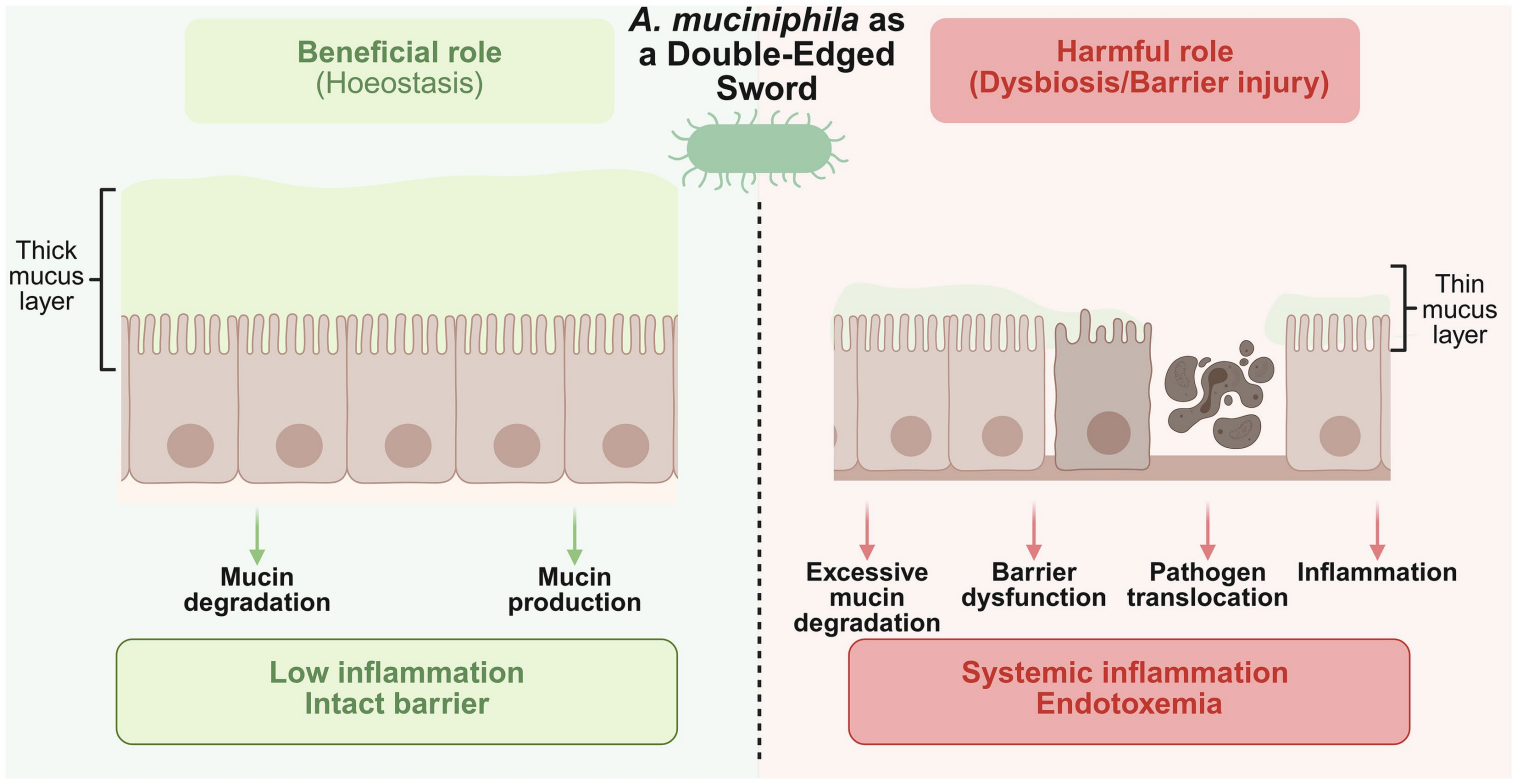
*Akkermansia muciniphila* as a double-edged sword.

*A. muciniphila* colonizes the intestinal mucus layer, where it degrades mucins to produce SCFAs and provides energy to the host. It also stimulates the secretion of additional mucins, thereby maintaining the thickness and integrity of the mucus layer (Ioannou et al., 2025b; Liu M. J. et al., 2022). However, in a model of radiation-induced intestinal injury, *A. muciniphila* grew markedly to impair the mucus layer by upregulating genes associated with mucin metabolism, allowing pathogen adhesion to epithelial cells and additional activation of pro-inflammatory macrophages (Figure 3) (Wang Y. et al., 2025). Yin et al. (2025) have reported that in a DOX-induced DIC model, the abundance of abnormally over-proliferative endogenous *A. muciniphila* is positively correlated with the degree of cardiac injury, and inhibition of the NLRP3 inflammasome reduces *A. muciniphila* abundance and alleviates cardiac dysfunction. This supports the pivotal regulatory role of the NLRP3 inflammasome in aggravating cardiac injury by increasing the abundance of *A. muciniphila*. Lin et al. (2025), on the contrary, have demonstrated the beneficial effects of an exogenous supplementation of *A. muciniphila*. This discrepancy may be attributed to the different DOX administration protocols used in the two studies. Yin et al. employed a lower cumulative dose (15 mg/kg) via short-term, high-frequency intravenous injection, potentially inducing acute intestinal inflammation and compensatory proliferation of *A. muciniphila*; in contrast, the latter adopted a higher cumulative dose (20 mg/kg) through long-term, low-frequency intraperitoneal injection, causing chronic mucosal damage, barrier disruption, and persistent depletion of *A. muciniphila*, thereby leading to the contrasting roles of *A. muciniphila*.

In 2016, a relevant study published in *Cell* demonstrated that under the stimuli of dietary fiber deprivation, the obvious increase in *A. muciniphila* (increased from ~20 to 40%) abundance induces the secretion of copious mucin-degrading enzymes and, in concert with other mucolytic microbiota, results in intestinal mucosal barrier disruption and a susceptibility to pathogenic infections (Desai et al., 2016). Furthermore, food intake was reduced in a cohort of 119 patients after hematopoietic cell transplantation (HCT), and 63 (53%) developed fever following severe neutropenia. This may be attributed to the decreased propionate concentration in response to intestinal caloric restriction, which reverses the inhibition of *A. muciniphila* metabolism and accelerates mucin degradation. The increased abundance of *A. muciniphila* eventually causes neutropenic fever by impairing the mucus barrier, facilitating bacterial antigen translocation, and triggering systemic inflammation (Schwabkey et al., 2022). Nevertheless, the mechanistic details underlying propionate-mediated inhibition of *A. muciniphila* metabolism are yet to be elucidated. In experimental models of HFD-induced obesity and dextran sulfate sodium (DSS)-induced colitis, a marked elevation in *A. muciniphila* abundance increased mucolytic activity, disrupted the physical integrity of the intestinal mucus barrier, and stimulated microbial infiltration. Consequently, Toll-like receptor 2 (TLR2) and nucleotide-binding oligomerization domain-containing protein 2 (NOD2)-induced pro-inflammatory signaling cascades are activated in intestinal epithelial cells (Larsen et al., 2021). Overall, despite the beneficial effects of *A. muciniphila* on intestinal homeostasis, it can also be a pro-inflammatory effector that harms intestinal barrier integrity in pathological states such as dietary fiber deprivation, DSS-induced colitis, and HFD-induced obesity. As emphasized by Luo et al. (2022a), *A. muciniphila* serves as a double-edged sword that not only possesses therapeutic advantages as a next-generation probiotic (NGP) but also presents context-dependent challenges.

## 6 Dietary patterns, medications, and baseline abundance influence the personalized therapeutics of *A. muciniphila* in tailoring CVDs

Both dietary and pharmaceutical factors influence the abundance of *A. muciniphila*. The proliferation of *A. muciniphila*, as promoted by a low-fiber diet, impairs intestinal barrier integrity (Desai et al., 2016). A randomized controlled trial involving overweight and obese subjects has demonstrated that a Mediterranean diet significantly increases the abundance of *A. muciniphila* within 8 weeks, which is independently associated with better metabolic parameters and milder inflammation (Tagliamonte et al., 2021). Seaweed and algae (e.g., kelp), as staples in the Korean diet, are rich in sulfated carbohydrates and *A. muciniphila* (Shang et al., 2017), potentially explaining the high sulfatase activity observed in *A. muciniphila* strains derived from the Korean population (Kim et al., 2022). Shearer et al.(Shearer et al., 2024) analyzed the association between gut microbiota and 12 commonly used medications in 134 patients with cardiometabolic diseases using multivariate linear regression. They found that beta-blockers and statins were significantly associated with a reduced abundance of *A. muciniphila*, whereas the combination of statins and non-steroidal anti-inflammatory drugs (NSAIDs) increased its abundance. In a rodent model of impaired glucose tolerance, metformin administration markedly elevated the abundance of *A. muciniphila* in the gut (Ermolenko et al., 2022). Consequently, a comprehensive assessment of dietary habits and pharmacotherapy history is of great significance in the management of CVDs, enabling individualized interventions targeting the dynamic regulation of *A. muciniphila* abundance and balancing the homeostatic environment.

A review published in the *New England Journal of Medicine* noted that glucagon-like peptide-1 (GLP-1) receptor agonists, such as semaglutide and tirzepatide, have been demonstrated in large-scale clinical trials to significantly reduce the risk of cardiovascular events in patients with type 2 diabetes and obesity (Rosen and Ingelfinger, 2026). In parallel, *A. muciniphila* can promote endogenous GLP-1 secretion through multiple pathways: the propionate it produces induces GLP-1 release via G protein-coupled receptor 41 (GPR41) and G protein-coupled receptor 43 (GPR43), whereas its secreted P9 protein can indirectly enhance GLP-1 secretion by improving glucose metabolism (Cani and Knauf, 2021). Wang et al. (2026) provided evidence from an environmental exposure perspective—polyethylene microplastic exposure significantly reduced *A. muciniphila* abundance in the mouse gut; decreased its metabolites myristic acid and phenylacetylglycine, thereby inhibiting the GLP-1 secretory pathway mediated by G protein-coupled receptor 120 (GPR120) and peptide transporter 1 (Pept1); and ultimately lowered plasma GLP-1 levels (Wang et al., 2026). In summary, *A. muciniphila* and its metabolites play a key role in modulating GLP-1 secretion; however, whether this bacterium has synergistic or other interactions with GLP-1 receptor agonist drugs remains unclear.

Notably, the therapeutic efficacy of *A. muciniphila* supplementation in individuals with overweight/obesity and type 2 diabetes was determined based on baseline intestinal abundance. In those with a low baseline abundance, *A. muciniphila* supplementation drives colonization in the intestinal mucus layer and benefits metabolic improvements, such as reducing body weight, blood sugar, and blood lipids, as well as improving energy metabolism. Conversely, no significant alterations in glycemic, lipid, and energy metabolism were detected in the high-abundance cohort, which are also validated in germ-free murine models (Zhang Y. et al., 2025). Therefore, we suggest that baseline abundance is a critical factor potentially influencing the therapeutic efficacy of *A. muciniphila* in CVDs, although clinical evidence requires further exploration.

## 7 Future perspectives

*A. muciniphila* acts as a beneficial commensal bacterium in the cardiovascular system in a dual manner. It directly protects against CVDs by enhancing intestinal barrier function and attenuating systemic inflammation and indirectly reduces cardiovascular risk by ameliorating metabolic disorders (e.g., obesity and diabetes). More notably, postbiotic forms of *A. muciniphila*, including pasteurized cells, the outer membrane protein Amuc_1100, and secreted membrane vesicles (MVs) (Natalia et al., 2020), likewise confer significant cardiovascular and metabolic benefits (Abraham et al., 2025; Salminen et al., 2021; Shaheen et al., 2025; Wu X. et al., 2025). This transcends the traditional scope of probiotics, paving the way for more flexible and safer formulation strategies for the clinical translation of *A. muciniphila*.

In the future, strain heterogeneity and dosing safety of *A. muciniphila* supplements are major issues that need to be resolved before clinical translation. A much finer strain-level classification of *A. muciniphila*, rather than species-level identification, can genetically and functionally identify trait characteristics for clinical applications (Hong et al., 2025; Luo et al., 2022a; Zhou et al., 2021). Well-characterized strains of *A. muciniphila,* specifically based on the target disease and host origin, are expected to benefit precise CVD management (Guo et al., 2017). For example, an *A. muciniphila* Group A strain harboring the *anSME* gene displays specific evolutionary advantages of high sulfatase activities and a potent ability to utilize sulfated mucins, allowing acceptable intestinal colonization and a strong competitive dominance (Kim et al., 2022). Amuc_1100, a core effector molecule of *A. muciniphila*, was administered to mice at a dose of 100 μg/day. The safety of high clinical dosing that far exceeds the physiological concentration of *A. muciniphila* in the gut requires further investigation (Wu X. et al., 2025).

Due to the low sensitivity of oral *A. muciniphila* supplements to gastric acid and oxygen (survival rate<0.02%), targeted delivery systems are urgently needed to optimize their clinical application. Dual-layer encapsulation strategies (e.g., pectin-carboxymethyl cellulose microcapsules and water-in-oil-in-water [W/O/W] double emulsions) can effectively shield *A. muciniphila* from gastric acid erosion, which increases the survival rate to 6.6% at pH 3.0 (van der Ark et al., 2017). This enables a colon-specific delivery of *A. muciniphila* that can promote colonization in the intestinal mucus layer and modulate the gut microbiota (Ta et al., 2024). Li et al. (2024) have developed a targeted nanodrug assembled from the *A. muciniphila* outer membrane protein Amuc_1100, fluorinated polyetherimide, and hyaluronic acid, allowing escape from enzymatic degradation and clearance *in vivo* and achieving the goal of minimizing chemotherapy-induced cardiotoxicity. In the future, more advanced delivery systems to safeguard colonization are needed to expand the glories of *A. muciniphila* in treating CVDs and other diseases.

## 8 Conclusion

As a representative next-generation probiotic, *A. muciniphila* demonstrates remarkable potential in the prevention and management of CVD owing to its superior intestinal colonization stability and favorable safety profile, which overcome the limitations of traditional probiotics. However, studies have also revealed that under specific pathological conditions, such as an impaired intestinal barrier or dysbiosis, *A. muciniphila* may shift from a protector to a potential risk factor, indicating that it should not be regarded as a simple “supplementation” but instead be applied according to precise strain selection, strict dosage control, and tailored intervention strategies. Currently, key technological advances, such as fermentation inactivation and microencapsulation, have broadened the applications of *A. muciniphila*. Therefore, *A. muciniphila* may serve as a novel microbial tool for CVD management.

## Abbreviations

AAA: Abdominal aortic aneurysm
*A. muciniphila*: *Akkermansia muciniphila*
AF: Atrial fibrillation
AmEVs: *A. muciniphila*-derived extracellular vesicles
ASR: Assimilatory sulfate reduction
*α*-SMA: Alpha-smooth muscle actin
AhR: Aryl hydrocarbon receptor
AMPK: adenosine monophosphate (AMP)-activated protein kinase
ApoE: Apolipoprotein E
Arg1: Arginase 1
ASCVD: Atherosclerotic cardiovascular disease
ATCC: American Type Culture Collection
CD4: Cluster of differentiation 4
CD14: Cluster of differentiation 14
CD206: Cluster of differentiation 206
CHF: Chronic heart failure
CK-MB: Creatine kinase-MB isoenzyme
CntA/B: Choline TMA-lyase
CPT1A: Carnitine palmitoyl transferase 1A
CREBH: Cyclic adenosine monophosphate responsive element-binding protein H
CITED2: Cbp/p300-interacting transactivator 2
CutC/D: Choline utilization protein C/D
CVD: Cardiovascular disease
DIC: Doxorubicin-induced cardiotoxicity
DOX: Doxorubicin
DSS: Dextran sulfate sodium
*E. coli*: *Escherichia coli*
EPAS1: Endothelial PAS domain-containing protein 1
EU: European Union
FMT: Fecal microbiota transplantation
*F. prausnitzii*: *Faecalibacterium prausnitzii*
Foxp3: Forkhead box P3
GLP-1: Glucagon-like peptide-1
GPR41: G protein-coupled receptor 41
GPR43: G protein-coupled receptor 43
GPR120: G protein-coupled receptor 120
HCT: Hematopoietic cell transplantation
HDL-C: High-density lipoprotein cholesterol
HFD: High-fat diet
HF: Heart failure
HFpEF: Heart failure with preserved ejection fraction
HFrEF: Heart failure with reduced ejection fraction
HTN: Hypertension
ICAM-1: Intercellular adhesion molecule-1
IL-10: Interleukin-10
IL-6: Interleukin-6
IL-1*β*: Interleukin-1 beta
IL-17A: Interleukin-17A
IPA: Indole-3-propionic acid
IRS-1: Insulin receptor substrate-1
IVIG: Intravenous immunoglobulin
JAK/STAT: Janus kinase / Signal transducer and activator of transcription
JNK: c-Jun N-terminal kinase
KD: Kawasaki disease
LDA: Linear discriminant analysis
LDH: Lactate dehydrogenase
LDL-C: Low-density lipoprotein cholesterol
LCWE: *Lactobacillus casei* cell wall extract
LPS: Lipopolysaccharide
LVEF: Left ventricular ejection fraction
LVFS: Left ventricular fractional shortening
miR-143/145: MicroRNA-143/145
miR-208a-3p: MicroRNA-208a-3p
MOMA-2^+^ Mφ: Monocyte/macrophage antigen-2-positive macrophages
MCP-1: monocyte chemoattractant protein-1
MOMA-2: Monocyte/macrophage antigen-2
NAM: Nicotinamide
NAD^+^: Nicotinamide adenine dinucleotide
NICD: Notch Intracellular Domain
NGP: Next-generation probiotic
NLRP3: NLR family pyrin domain containing 3
NOD2: Nucleotide-binding oligomerization domain-containing protein 2
NOVA1: Neuro-oncological ventral antigen 1
NSAIDs: Non-steroidal anti-inflammatory drugs
NNMT: Nicotinamide N-methyltransferase
MVs: Membrane vesicles
PAH: Pulmonary arterial hypertension
PASMCs: Pulmonary arterial smooth muscle cells
PE: Preeclampsia
PGC1α: Peroxisome proliferator-activated receptor gamma coactivator 1-alpha
PPARα: Peroxisome proliferator-activated receptor alpha
Pept1: Peptide transporter 1
qPCR: Quantitative polymerase chain reaction
RAAS: Renin-angiotensin-aldosterone system
RORγt^+^: Retinoic acid receptor-related orphan receptor gamma t-positive
SCFAs: Short-chain fatty acids
SHRs: Spontaneously hypertensive rats
SIRT3: Sirtuin 3
SM22α: Smooth muscle protein 22-alpha
STAT3: Signal transducer and activator of transcription 3
TC: Total cholesterol
Th17: T helper 17 cells
TLR: Toll-like receptor
TLR2: Toll-like receptor 2
TLR2/4: Toll-like receptor 2/4
TMA: Trimethylamine
TMAO: Trimethylamine N-oxide
TNF-α: Tumor necrosis factor-alpha
Tregs: Regulatory T cells
VSMC: vascular smooth muscle cell
Wnt/β-catenin: Wingless-related integration site / beta-catenin
W/O/W: Water-in-oil-in-water
YeaW/X: YeaW/X enzyme
ZO-1: Zonula occludens-1
